# Representation of Dormant and Active Microbial Dynamics for Ecosystem Modeling

**DOI:** 10.1371/journal.pone.0089252

**Published:** 2014-02-18

**Authors:** Gangsheng Wang, Melanie A. Mayes, Lianhong Gu, Christopher W. Schadt

**Affiliations:** 1 Climate Change Science Institute, Oak Ridge National Laboratory, Oak Ridge, Tennessee, United States of America; 2 Environmental Sciences Division, Oak Ridge National Laboratory, Oak Ridge, Tennessee, United States of America; 3 Biosciences Division, Oak Ridge National Laboratory, Oak Ridge, Tennessee, United States of America; J. Craig Venter Institute, United States of America

## Abstract

Dormancy is an essential strategy for microorganisms to cope with environmental stress. However, global ecosystem models typically ignore microbial dormancy, resulting in notable model uncertainties. To facilitate the consideration of dormancy in these large-scale models, we propose a new microbial physiology component that works for a wide range of substrate availabilities. This new model is based on microbial physiological states and the major parameters are the maximum specific growth and maintenance rates of active microbes and the ratio of dormant to active maintenance rates. A major improvement of our model over extant models is that it can explain the low active microbial fractions commonly observed in undisturbed soils. Our new model shows that the exponentially-increasing respiration from substrate-induced respiration experiments can only be used to determine the maximum specific growth rate and initial active microbial biomass, while the respiration data representing both exponentially-increasing and non-exponentially-increasing phases can robustly determine a range of key parameters including the initial total live biomass, initial active fraction, the maximum specific growth and maintenance rates, and the half-saturation constant. Our new model can be incorporated into existing ecosystem models to account for dormancy in microbially-driven processes and to provide improved estimates of microbial activities.

## Introduction

Ecologically-important processes such as soil organic carbon and nutrient cycling largely depend on the active fraction of microbial communities [1]. At any given time in a given environment, microorganisms can be in active, dormant, or dead states [2]. Dormancy is considered an evolutionary strategy designed to maintain the genetic code until conditions improve to allow replication [3]. When environmental conditions are unfavorable for growth, e.g., resource limitation, microbes may enter a reversible state of low to zero metabolic activity to alleviate the loss of biomass and metabolic functions [4], [5]. The maintenance coefficient (i.e., maintenance cost of C per unit microbial biomass C per unit time) can be two to three orders of magnitude lower in dormant microbes than in metabolically active microbes [6], [7]. Many soils have slow organic matter turnover rates with seasonal changes in substrate supply, temperature, and moisture. The complexity of soils in space and time may result in uneven distributions of multiple potentially limiting resources, leading to significant rates of dormancy even when some resources are abundant. When spatial and temporal complexity is combined with differential resource partitioning among species in a community, high rates of dormancy could be a prominent feature in soil systems. Thus it is essential to understand dormancy in order to more accurately predict how active microorganisms contribute to ecosystem processes such as decomposition and nutrient turnover [1].

A complicating factor in studying microbial dormancy is that no single approach can be easily employe to simultaneously measure individual microbial states (active, dormant or dead), and a combination of different techniques is required. Differential staining is often used to segregate physiological states with direct microscopic counting of bacteria and fungi. ‘Life-indicating’ stains that require the presence of ‘standard’ physiological abilities, such as the esterase activity needed for fluorescein diacetate cleavage, may distinguish active from dormant+dead cells [8]. When combined with general-purpose stains, these strains can distinguish dormant cells by difference [9]. Combining membrane-permeant with membrane-impermeant nucleophilic stains (e.g., SYTO-9 and propidium iodide respectively) may distinguish live from dead, but not active from dormant [10], [11]. Active microbes may or may not be ‘viable’ with common culture-based techniques, which complicates classification and measurement of dormancy phenomena [5]. Methods such as direct plating, serial dilution and most probable number (MPN) techniques will not distinguish between active and dormant organisms [12]. Substrate Induced Respiration (SIR) or Substrate Induced Growth Response (SIGR) method [13], [14] can distinguish active and dormant communities if growth respiration curves are modeled (using initial exponentially-increasing respiration); however, the technique often needs to be combined with microscopy or chloroform fumigation/extraction in order to obtain total live microbial biomass [15], [16].

Despite limitations in distinguishing active, dormant and dead microbial biomass, abundant evidence indicates that the majority of environmental microorganisms in a given community may be dormant under natural conditions [1], [17]. Alvarez *et al*. [18] reported that only 3.8–9.7% of the total biomass is active in a Typic Argiudoll soil from the Argentinean Pampa. Khomutova *et al*. [19] showed that the fraction of active microbial biomass ranged from 0.02% to 19.1% in the subkurgan paleosoils of different age and 9.2–24.2% in modern background soils. Microbial biomass measured through SIR or SIGR is thought to reflect only the active portion because the maintenance respiration of dormancy biomass is negligible in the initial exponentially-increasing phase [13], [16], [20]. Through a mathematical analysis of respiration curves, Van de Werf & Verstraete [21] examined 16 soils and found that 4–49% of the total biomass was in an active state; and the active component in undisturbed natural ecosystems (18.8±8.8%, mean±standard deviation) was about 70% of that in arable agricultural soils (25.7±14.8%). Stenström *et al*. [22] showed that the fraction of active biomass typically varied from 5% to 20% in soils without recent addition of substrates. Lennon & Jones [5] found much lower active fractions in soils (18±15%) than in marine (65±19%) and fresh (54±11%) water environments. From the above studies it seems conservative to extrapolate that the active fraction is very likely below 50% of live microbes under most natural soil conditions.

Microbially-mediated processes have been incorporated into ecosystem models [23]–[28] although continued development is still required to bring microbial processes into global climate models [29]–[31]. However, these recent models do not consider physiological state changes and assume that measures of microbial biomass constitute the active biomass. The exclusion of dormancy from the microbially-driven ecosystem processes could result in incorrect estimates of total live microbial biomass, which further leads to deficiencies in model parameterization and predictions of soil organic carbon and nutrients.

Generally, there are two strategies to represent physiological states in microbial-ecology models: one is to explicitly separate the total live biomass into two pools, i.e., active and dormant [4], [32]; the other is to directly regard the active fraction (i.e., ratio of active biomass to total live biomass) as a state variable [33], [34]. Both of these two approaches predict the total live biomass, active and dormant biomass, and the flux between the active and dormant components. Apparently the introduction of the ‘active fraction’ as a state variable in the latter approach simplifies the model structure since the adaptive variation of microbial composition might be represented by one single variable (active fraction) [34], [35]. However, another state variable indicating the microbial biomass pool size (e.g., total live biomass, active biomass or dormant biomass) is still essential for ecosystem modeling since the carbon and nutrient fluxes are pool-size dependent. For example, if we define active fraction and total microbial biomass as state variables, the active and dormant biomass could be determined by them, and the net flux between active and dormant fractions and other related fluxes could also be computed according to the active and dormant biomass constrained by mass balance. The above-mentioned modeling efforts have shown that adequate representation of dormancy and the transitions between the dormant and active states is crucial for modeling important microbially-mediated ecosystem processes.

Here, we review state-of-the-art microbial dormancy modeling approaches and discuss the rationales of these models with a focus on transformation processes between active and dormant states. We propose an improved synthetic microbial physiology model based on accepted assumptions and examine the model behavior with theoretical and experimental analyses. In this paper, the ‘total microbial biomass’ refers to the ‘total live microbial biomass’ unless otherwise stated. Our objective is to clarify the applicability of existing microbial dormancy models and provide a new theoretical basis for representing microbial activity and dormancy in ecosystem models.

## Dormancy In Microbial Models

### Transformation between active and dormant states

**Figure 1:**
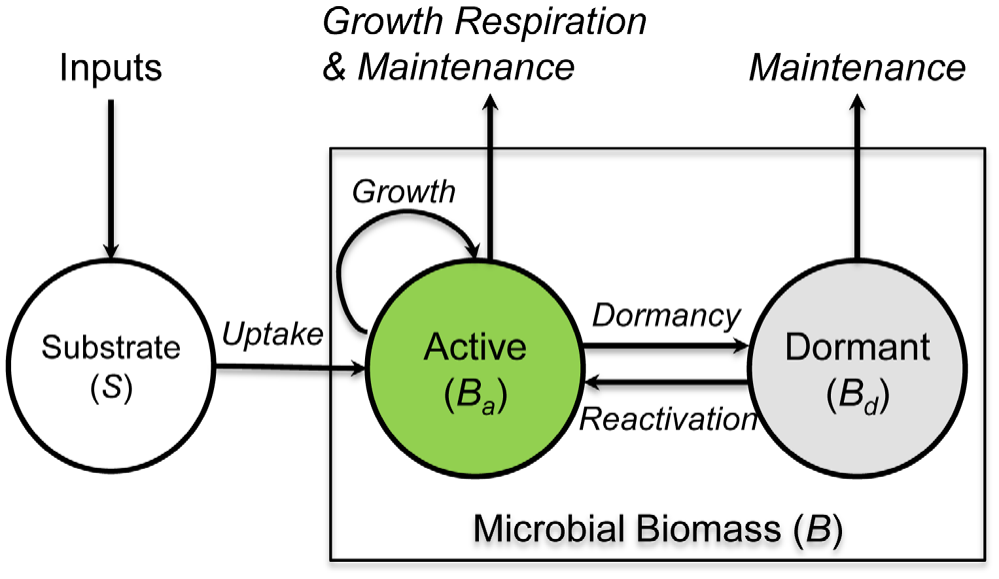
Active and dormant microbial biomass pools in microbial physiology models (modified from Fig. 2 in Lennon & Jones, 2011).

Although Buerger *et al*. [36] argued that dormant microbial cells could reactivate stochastically and might be independent of environmental cues, environmental factors such as substrate availability are often thought to control the transformation between active and dormant states [5]. Most models (see Appendix S1) distinguish the active biomass pool from the dormant pool and define them as two state variables (*B_a_* and *B_d_*) (Fig. 1). Only active microbes (*B_a_*) can uptake substrate and produce new cells. The connection between the active and dormant states is a reversible process including two directional sub-processes, i.e., dormancy (from active to dormant) and reactivation (or resuscitation, from dormant to active). Losses from active biomass include growth respiration and maintenance (maintenance respiration, mortality, enzyme synthesis, etc.) [23]. Dormant microbes still require energy for maintenance and survival although at a lower metabolic rate [5].

The net transformation rate (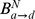) from active to dormant state is the difference between the flux from active to dormant (*B_a_*_→*d*_) and the flux from dormant to active state (*B_d_*_→*a*_), i.e., 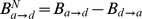. The models of Hunt [37] (Equation S1-1 in Appendix S1) and Gignoux *et al*. [38] (Equation S1-2 in Appendix S1) directly formulate the net flux (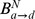) without explicit components for *B_a_*_→*d*_ and *B_d_*_→*a*_. The direction of the net flux depends on the maintenance requirement relative to the substrate availability. If the available substrate is less than the maintenance requirement, there is a positive net flux from active to dormant pool, and vice versa. In addition, Hunt [37] assumed a ‘buffer zone’ for the change of states: when the maintenance requirement surpasses the substrate supply but the deficit is within a small fraction (1% d^−1^) of *B_a_*, there is no flux between the two states.

Some models define rates for both dormancy and reactivation. In the model of Ayati [39] (Equation S1-3 in Appendix S1), the dormant rate (*γ_a_*_→*d*_) increases with declining substrate concentration, and the reactivation (*γ_d_*_→*a*_) only occurs when substrate concentration is higher than the half-saturation constant (*K_s_*). Konopka [32] modified the potential rates for dormancy and reactivation by the relative growth rate (*μ*/*μ*_max_, ratio of true specific growth rate to maximum specific growth rate), i.e, the two rates are multiplied by (1-*μ*/*μ*_max_) and *μ*/*μ*_max_, respectively (Equation S1-4 in Appendix S1). Similarly, Jones & Lennon [40] postulated two complementary rates (1-*R* and *R*) for dormancy and resuscitation (Equation S1-5 in Appendix S1).

Two other models also explicitly formulate the two conversion rates between states but do so using concepts of probability. Bär *et al*. [41] used two complementary factors (1-*J* and *J*) to represent the probability for the transition between active and dormant state in addition to an identical potential rate constant for the two processes (Equation S1-6 in Appendix S1). The conceptual model of Locey [42] applies a deterministic dormant rate and a stochastic resuscitation rate (Equation S1-7 in Appendix S1). The potential resuscitation rate is modified by (1-*p*), where *p* is the probability that a disturbance in the active pool will result in the immigration of one individual from the metacommunity. The probability (*J*) in Bär *et al*. [41] is explicitly calculated from the environmental cues (e.g., soil moisture), while the cause of the probability (*p*) in Locey [42] is not elucidated.

### Switch function model

In addition to the dormancy and reactivation processes, a key concept in the model developed by Stolpovsky *et al*. [4] is ‘switch function’ (Equation S1-8 in Appendix S1). The switch function (*θ*) determines the fraction of active cells taking up dissolved organic carbon (DOC). This function refers to the growth fraction in active biomass (*B_a_*) that consumes substrate and thus is not the same as the active fraction (*r*) in total biomass (*B*). Furthermore, the dormancy and reactivation fluxes are set to be proportional to (1-*θ*) and *θ*, respectively. *θ* is formulated by the Fermi-Dirac statistics [4]. Another feature of this model is the consideration of ‘depth’ of dormancy in reactivation, where the reactivation rate is negatively dependent on the duration of dormancy. The switch function model includes at least 15 model parameters and it is difficult to compute the Gibbs energy change of the oxidation of DOC [4].

The switch function (*θ*) sets it apart from the conventional Michaelis-Menten (M-M) or Monod kinetics because of its new perspective of thermodynamics. According to the M-M kinetics [43], the substrate saturation level represents the fraction of enzyme-substrate complex (*ES*) in active enzyme (*E*_0_), where the substrate saturation level is formulated by *S*/(*K_s_* + *S*) with *S* and *K_s_* being the substrate concentration and the half-saturation constant [43]. When the M-M kinetics is applied to describe microbial uptake of substrate, the substrate (or combined with TEA) saturation level (i.e., *μ*(*S*, *TEA*) in Equation S1-8a of Appendix S1) is a measure of the actively growing fraction in the active microbial community. The switch function is also determined by the saturation levels of substrate and terminal electron acceptor (TEA), i.e., *μ*(*S*, *TEA*) (Equation S1-8e in Appendix S1). Mathematically the inclusion of both the switch function (*θ*) and the M-M kinetics (i.e., *μ*(*S*, *TEA*)) might result in double counting of the impact of substrate and TEA. We would recommend using the switch function (*θ*) to modify the microbial uptake rate if the Gibbs energy change of the oxidation of substrate (Δ*G*) is tractable and the thermodynamic threshold (*G*_0_) and the steepness of the step function (*st*) are identifiable.

### Physiological state index models

As an alternative to models with two microbial biomass pools (i.e., active and dormant), a further state variable indicating the dormant or active fraction in total biomass has been proposed. Wirtz [44] developed a simple index (*r_d_* = 0.5–1.0) representing the dormant microbial biomass as a fraction of the steady-state total biomass (*B_stat_*) under the condition of *B_d_* << *B_a_*. In case of a net loss of total biomass (*dB*/*dt*<0), the dormant biomass *B_d_* = *B_stat_*· *r_d_*; otherwise (*dB*/*dt*>0), *B_d_* = *B_stat_*·(1−*r_d_*). This model has a sudden change of dormant biomass at the transition point (i.e., *dB*/*dt* = 0) since *r_d_*>0.5.

Different from the dormant index of Wirtz [44], the concept of an active index (i.e., index of physiological state) of soil microbial community has been employed in soil carbon and nutrient cycling models [33], [34]. The index of physiological state (*r*), referring to the activity state, is often defined as the ratio of metabolically active microbial biomass to the total soil microbial biomass [22], [33], [34].

In the Synthetic Chemostat Model (SCM), the rate of change of the state variable *r* is described as follows [33], [45]: 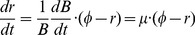
(1)with 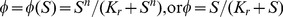
(2)where *r* = *B_a_*/*B*, representing the fraction (hereinafter referred to as ‘active fraction’) of active biomass in total biomass; *μ* is the specific growth rate of total biomass; 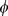 denotes the saturation level of substrate (*S*); the simple power (*n* = 1) has been widely used [35] and, in this case (*n* = 1), *K_r_* is called the half-saturation constant.

Blagodatsky & Richter [34] used the expression 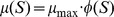 in their model development. This expression was not derived in the original definition of the specific growth rate (see Equation 3) by Panikov [45] and because its validity cannot be inferred, the concepts will not be addressed here.

According to Panikov's derivation [45], the specific growth rate (*μ*) follows the general definition [46], [47]:
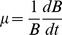
(3)


Based on Equations 1 and 3, we can derive (see Equation S2-1 in Appendix S2):
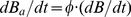
(4)

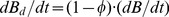
(5)


We find that the model described by Equation 1 is not applicable under low substrate availability, as described below. Generally, the rates of change in biomass pools (*B*, *B_a_*, and *B_d_*) can be expressed as
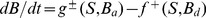
(6)

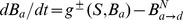
(7)

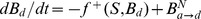
(8)where 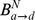 denotes the net dormancy flux; *g* ^±^(*S*, *B_a_*) is a function that represents the difference between the substrate uptake and the maintenance requirements of *B_a_*, i.e., the net growth of *B_a_*; and *f* ^+^(*S*, *B_a_*) is a function denoting the maintenance and survival energy costs of *B_d_*. The superscript ‘±’ in *g* ^±^ indicates the function value of *g* could be positive at high *S* or non-positive when the substrate uptake cannot satisfy the maintenance requirements of *B_a_* at low *S*. The superscript ‘+’ in *f* ^+^ implies *f*≥0. Note that the function *f* ^+^(*S*, *B_a_*) is not necessarily dependent on *S* [4].

From Equations 4, 6 and 7, we can obtain
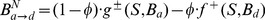
(9)


The two terms in the right side of Equation 9 may be regarded as the conversion of *B_a_* to *B_d_* (i.e., *B_a_*_→*d*_) and the transformation of *B_d_* to *B_a_* (i.e., *B_d_*_→*a*_), respectively. At high *S* resulting in *g*≥0, Equation 9 may be one of the possible expressions for *B_a_*_→*d*_ and *B_d_*_→*a*_. However, at low *S* leading to *g*<0 and *B_a_*_→*d*_<0, i.e., no active cells become dormant under insufficient substrate, which is inconsistent with the strategy of dormancy for microorganisms when faced with unfavorable environmental conditions [5].

Based on the above analysis, we conclude that the physiological state index model (Equation 1) needs to be improved. In other words, the empirical assumption that the steady state active fraction (*r^ss^*) approaches the substrate saturation level (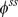) may not be necessary because this assumption could lead to impractical flux (Equation 9) between dormant and active states under low substrate availability.

### A Synthetic Microbial Physiology Model

Based on the aforementioned review and analysis, we have developed a synthetic microbial physiology model component relating to substrate availability. As indicated by Fig. 1, the growth and maintenance functions of active microbes (*B_a_*) are characterized by the maximum specific growth rate (*μ_G_*) and maintenance rate (*m_R_*); whereas the dormant microbes (*B_d_*) cost energy to maintain their basic cellular functions at a much lower specific maintenance rate (denoted by *β*·*m_R_*, where *β*<1) [48].

### General assumptions

First we define the substrate saturation level (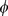) as
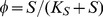
(10)


where the parameter *K_s_* is the half saturation constant for substrate uptake as indicated by the M-M kinetics [43].

Based on the above review of existing dormancy models, the following assumptions are accepted in our new model: (1) the dormancy rate is proportional to the active biomass and the reactivation rate is proportional to the dormant biomass, i.e., 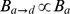 and 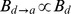; (2) under very high substrate concentration (*S*>>*K_s_*), 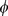
→1, *B_a_*_→*d*_→0 and *B_d_*_→*a*_≥0; (3) under very low substrate (*S*<<*K_s_*), 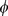
→0, *B_a_*_→*d*_≥0 and *B_d_*_→*a*_→0; (4) based on the assumptions (1–3), we derive that 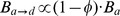 and 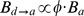; (5) further we assume that the maximum specific maintenance rate for active microbes (*m_R_* with units of h^−1^) controls both transformation processes since the maintenance energy cost is the key factor regulating the dormancy strategy [5], [37], [38]. As a result we postulate that
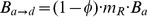
(11a)

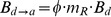
(11b)


## Model Description

Equations 11a and 11b only describe the transformation between the active and dormant states. They need to be linked to a microbial growth and maintenance model for depicting microbial dynamics. Our recent work to develop the Microbial-ENzyme-mediated Decomposition (MEND) model [23] suggested that it might be adapted to serve this purpose due to its focus on microbial processes for which we have developed a firm theoretical basis [47], [49]. Combining Equations 11a and 11b with the MEND model [23], [47], we express the microbial physiology component (see Fig. 1) as a group of differential equations
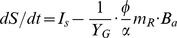
(12a)

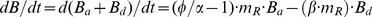
(12b)

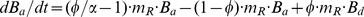
(12c)

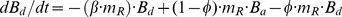
(12d)where *t* is the time scale; 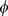 is defined by Equation 10; *I_s_* is the input to substrate pool; *Y_G_* is the true growth yield; *m_R_* denotes the specific maintenance rate at active state (h^−1^); *α* = *m_R_*/(*μ_G_*+*m_R_*) is the ratio of *m_R_* to the sum of maximum specific growth rate (*μ_G_*) and *m_R_*, 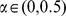 since usually *m_R_*≤*μ_G_*; and *β* (0–1) is the ratio of dormant maintenance rate to active maintenance rate, i.e., (*β m_R_*) denotes the maximum specific maintenance rate at dormant state.

In summary, there are five parameters (*α*, *β*, *m_R_* or *μ_G_*, *Y_G_*, *K_s_*) in the proposed model (hereinafter referred to as the MEND model). From Equations 12b and 12c, we can derive the change rate of active fraction (*r*) (see Equation S2-2 in Appendix S2)
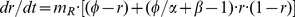
(12e)


This equation for *r* is more complicated than Equation 1 but still practical, given currently available data. Additionally, it implies that *r* needs not approach 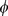 at steady state in our model, whereas *r*≡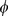 at steady state is required by the model of Panikov [45].

### Steady state analysis

**Figure 2:**
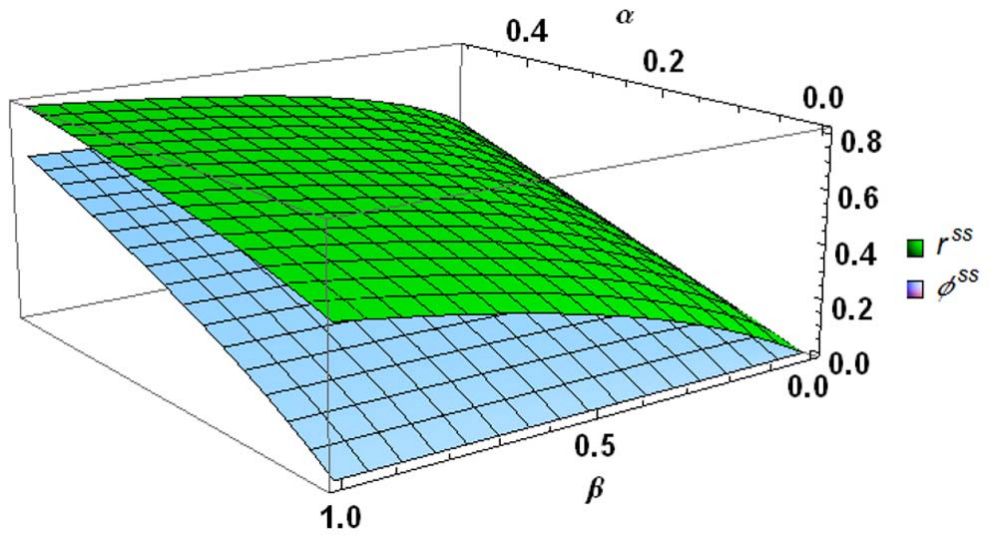
Steady state active fraction (*r^ss^*) and substrate saturation level (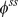) as a function of *α* and *β*; *α*  =  *m_R_*/(*μ_G_*+*m_R_*), *μ_G_* and *m_R_* (h^−1^) are maximum specific growth rate and specific maintenance rate for active microbial biomass, respectivly; *β* denotes the ratio of dormant specific maintenance rate to *m_R_*.



Assuming the input (*I_s_*) is time-invariant, we can obtain the steady state solution to the above new MEND model (see Equations S2-3(a–e) in Appendix S2). Fig. 2 shows the steady state active fraction (*r^ss^*) and substrate saturation level (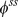) as a function of the two physiological indices, i.e., *α* (0–0.5) and *β* (0–1). Both *r^ss^* and 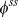 positively depend on *α* and *β* and *r^ss^*≥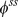 for any combinations of *α* and *β*. If we consider two extreme values of *β*→0 or *β*→1, the *r^ss^* and 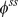 (see Equations S2-4 and S2-5 in Appendix S2) can be simplified to

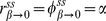
(13a)

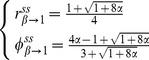
(13b)


Equation 13 and Fig. 2 indicate that: (1) the steady state active fraction (*r^ss^*) is equal to 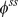 and they are identical to *α* = *m_R_*/(*μ_G_*+*m_R_*) only under the condition of *β*→0; (2) the upper bound of *r^ss^* is approximately 0.8 at *α*→0.5 and *β*→1; and (3) with *α*≤0.5, the maximum *r^ss^* is ca. 0.5 if the magnitude of *β* is around 0.001–0.01 [6]. This threshold value (0.5) of *r^ss^* is a reasonable estimate that can explain how the measured active fraction of microbes in undisturbed soils is usually considerably less than the total biomass [5], [21], [22].

### Model simplification under sufficient substrate condition

As mentioned in the Introduction, SIR or SIGR method can distinguish active from dormant composition and the data from these experiments have been widely used to estimate the active microbial biomass and the maximum specific growth rate [13], [14]. The simplification of the microbial model under excess substrate has also been employed to estimate maximum specific growth rate (*μ_G_*), active microbial biomass (*B_a_*), and/or total microbial biomass (*B*) using the SIR or SIGR data [1], [13], [35]. Here we show the simplification of our model (Equation 12) for conditions appropriate to SIGR or SIR experiments, e.g., the short-term period of exponentially-increasing respiration of active biomass following substrate addition. We will test our reduced and full model with the SIGR data of Colores *et al*. [13] in the next section.

Under sufficient substrate (i.e., *S*>>*K_s_* in Equation 10 thus 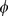
→1), Equations 12(a–e) can be simplified and integrated for initial conditions, i.e., *S* = *S*_0_, *B* = *B*_0_ and *r* = *r*_0_ at *t* = 0 (see Equations S2-6 and S2-7 in Appendix S2):
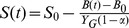
(14a)

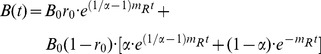
(14b)

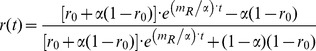
(14c)


The CO_2_ production rate, *v*(*t*), during the exponential growth stage is derived as an explicit function of *t* (see Equation S2-7d in Appendix S2): 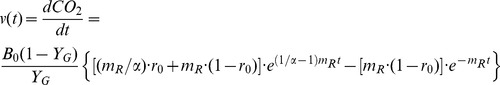
(14d)


The respiration rate, *v*(*t*), is associated with two exponential items, i.e., 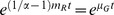
and 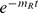. Considering an extreme case that *m_R_*<<*μ_G_* (i.e., 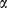
→0), Equations 14(b–d) can be further simplified to Equations S2-8(b–d) (see Appendix S2). Equations S2-8b and S2-8c (denoting *B*(*t*) and *r*(*t*), respectively) are similar to Equations 11 and 10 in Panikov & Sizova [35], respectively. However, Equation S2-8d (for *v*(*t*)) is different from Equation 13 of Panikov & Sizova [35], where a constant ‘*A*’ was added to the exponential. Equation S2-8d is identical to Equation 7 derived for SIGR experiments in Colores *et al*. [13].

Panikov & Sizova [35] used their Equation 13 to fit respiration rates during the exponentially-increasing (i.e., no substrate limitation) phase (see Fig. 2 in Panikov & Sizova [35] for data and curve fittings). However, these data are based on glucose-induced respiration that includes both basal respiration of native SOC and respiration due to the addition of glucose [13]. The basal respiration rate may be regarded as a constant in certain cases (see Colores *et al*. [13] and data in Fig. 1 of Blagodatsky *et al*. [50]). The constant ‘*A*’ representing the basal respiration rate was included in Equation 13 of Panikov & Sizova [35] in order to fit the combined respiration from the addition of glucose and basal respiration. However, this constant ‘*A*’ cannot be derived from such governing equations as Equations S2-6(a–c) (see Appendix S2) that assume respiration is the sole result of substrate addition. In other words, the equations do not include basal respiration. Certainly, our predicted respiration could include basal respiration as long as (i) a basal respiration rate is added to Eq. 14d *ad hoc* or (ii) Equations S2-6(a–c), or more commonly Equations 12(a–e), are linked to a soil organic matter (SOM) decomposition model, which can produce decomposed native soil C in addition to the respiration of substrate addition. Because Equation 13 of Panikov & Sizova [35] is not linked to a native C decomposition model, fitting the model to combined native C and substrate respiration data is not appropriate.

### Model test I: substrate-induced respiration

In this section, we used the respiration data from ^14^C-labeled glucose SIGR experiments by Colores et al. [13] to calibrate our MEND model. The respiration data only represented the CO_2_ production from the added substrate and did not include basal respiration from the native C.

**Figure 3:**
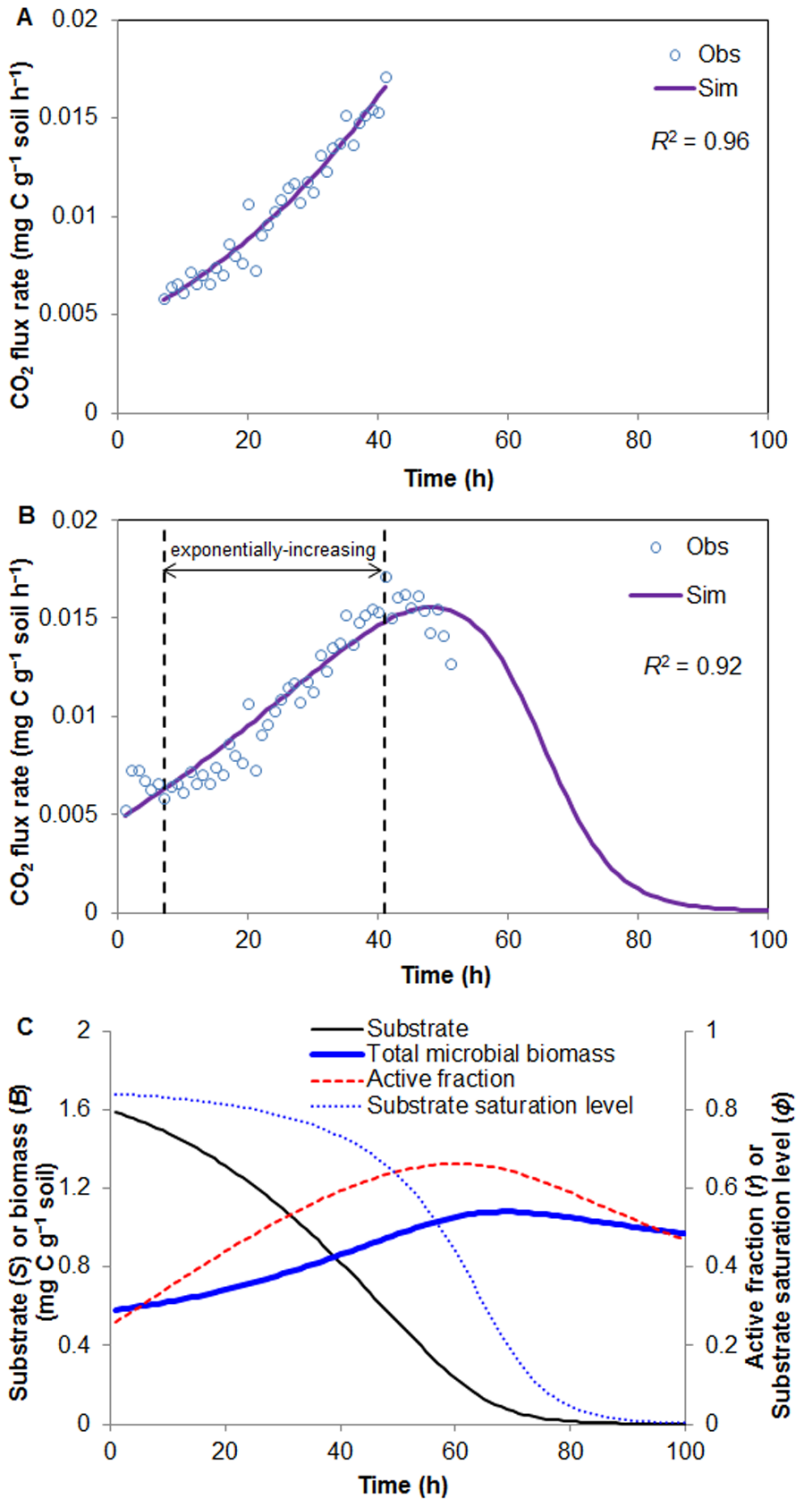
MEND model simulations against the respiration rates due to added ^14^C-labeled glucose in Colores et al. [**13**]. (a) Fitting of the respiration rates in the exponentially-increasing phase using Equation 14, ‘Obs’ and ‘Sim’ denote observed and simulated data, respectively. (b) Fitting of the respiration rates in both exponentially-increasing and non-exponentially-increasing phases using Equation 12. (c) Simulated substrate (*S*), total live microbial biomass (*B*), active fraction (*r*) and substrate saturation level (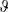) based on Equation 12.

First we employed Equation 14d to fit the respiration rates during the exponentially-increasing stage and the result is shown in Fig. 3a (see original data in Fig. 3 of Colores et al. [13]). The true growth yield (*Y_G_*) was set to 0.5 according to Colores *et al*. [13]. There are four undetermined parameters (*B*_0_, *r*_0_, *μ_G_*, *α*) in Equation 14d (with *m_R_*  =  *μ_G_ ·α*/(1−*α*)). We found that only the maximum specific growth rate (*μ_G_*) could be determined with high confidence (coefficient of variation (CV) = 5%) from the exponentially-increasing respiration rates. The CVs of the other three optimized parameters (*B*_0_, *r*_0_, *α*) were as high as 55–77% (Table 1). However, the initial active microbial biomass (*B_a0_*  =  *B*_0_×*r*_0_) had a lower uncertainty (CV = 20%) compared to *B*_0_ and *r*_0_. The above results indicate that the exponentially-increasing respiration rates can only be used to obtain *μ_G_* and *B_a0_*.

**Table 1:** MEND model parameters values used for simulation of respiration rates due to added ^14^C-labeled glucose in Colores et al. [13].

Parameter	Exponentially-increasing respiration*			All data†			Description
	Mean	SD‡	CV§	Mean	SD	CV	
*B*_0_	0.504	0.279	55%	0.525	0.080	15%	Initial microbial biomass, (mg C g^−1^ soil)
*r*_0_	0.394	0.263	67%	0.285	0.064	23%	Initial active fraction
*μ_G_*	0.027	0.001	5%	0.030	0.001	3%	Maximum specific growth rate (h^−1^)
*α*	0.185	0.142	77%	0.228	0.031	13%	*m_R_*/(*μ_G_*+*m_R_*), *m_R_* is maximum specific maintenance rate for active microbes (h^−1^)
*K_s_*	—	—	—	0.275	0.038	14%	Half-saturation constant for substrate (mg C g^−1^ soil)
*β*	—	—	—	0.025	0.019	76%	Ratio of dormant maintenance rate to *m_R_*
*Y_G_*	0.5	—	—	0.5	—	—	True growth yield, constant
*B_a0_*	0.135	0.027	20%	0.145	0.004	3%	Initial active biomass (mg C g^−1^ soil), calculated by *B*_0_×*r*_0_

*Only the respiration rates during exponentially-increasing phase are used.

† All data including both exponentially-increasing and non-exponentially-increasing respiration.

‡ SD: standard deviation.

§ CV: Coefficient of variation.

We then conducted numerical simulations in terms of all data including both exponentially-increasing and non-exponentially-increasing respiration rates (Fig. 3b). The non-exponentially-increasing respiration rates include the lag period before the exponentially-increasing phase and the respiration at longer times after the rates cease to increase exponentially [13]. The latter phase is likely because of the substrate saturation levels (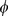) become limiting to respiration. We used Equations 12a, 12b, 12e and the corresponding expression for CO_2_ flux rate, to allow the substrate saturation level (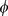) to change with time. Additionally, we used the ranges of *μ_G_* determined above. We used the SCEUA (Shuffled Complex Evolution at University of Arizona) algorithm [51], [52] to determine model parameters. The SCEUA is a widely used stochastic optimization algorithm for calibrating hydrological and environmental models [51].

When exponentially-increasing and non-exponentially-increasing data are included together, the CVs of all parameters (*B*_0_, *r*_0_, *μ_G_*, *α*, *K_s_*, *β*) are within 25% except *β* with a high CV of 76% (Table 1). The optimized *μ_G_* values (0.030±0.001 h^−1^) are almost the same as obtained by Colores *et al*. [13]. Model estimates of *α* = 0.228±0.031 indicate that the maximum specific maintenance rate of active microbes (*m_R_*) is about 30% of *μ_G_* and thus cannot be ignored. The initial active biomass (*B_a0_*) is 0.145±0.004 mg C g^−1^ soil (Table 1), which is lower than the values (0.194±0.004 mg C g^−1^ soil) using the SIGR method [13]. This is likely due to the inclusion of maintenance respiration (characterized by *m_R_*, see Equation 14d) in our model even for the exponentially-increasing stage; thus a lower *B_a0_* could produce similar CO_2_ flux to the case with higher *B_a0_* that does not include the contributions from maintenance respiration. Our results also show that the initial active fraction (*r*_0_) is 28.5±6.4% and *β* is 0.025±0.019. The magnitude of *β* is comparable to the estimation by Anderson & Domsch [6], [7]. In addition, the half-saturation constant (*K_s_*) was estimated as 0.275±0.038 mg C g^−1^ soil, which is very close to the values derived from 16 soils by Van de Werf & Verstraete [21]. This *K_s_* value indicates the substrate saturation level (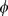) is higher than 0.7 before the transition from exponentially-increasing to non-exponentially-increasing phase (Fig. 3c). The changes of substrate (*S*), total microbial biomass (*B*) and active fraction (*r*) with time are also shown in Fig. 3c. In conclusion, the five parameters (*B*_0_, *r*_0_, *μ_G_*, *α*, *K_s_*) can be effectively determined using both exponentially-increasing and non-exponentially-increasing respiration rates, whereas *β* may also be determined but with a relatively high uncertainty (CV = 76%) than the other parameters.

Through this experimental analysis, we identified the need for isotopic data to discriminate between basal and substrate-induced respiration. We also discovered that the exponentially-increasing period due to substrate addition can be used to identify only a select set of model parameters (i.e., *μ_G_* and *B_a0_*) as also demonstrated by the method of Colores *et al*. [13]. These parameters, however, can be further applied to longer-term respiration experiments to enable fitting to obtain the remainder of model parameters by using our MEND model. Thus, we have found a new and unique solution to identify different parameters as a function of time, and to effectively use isotopic labeling to yield a specific set of model parameters.

### Model test II: intermittent substrate supply

In order to further validate this additional physiological component in the MEND model, we also tested it against a laboratory experimental dataset with intermittent substrate supply [4]. In addition to the substrate, another limiting factor (i.e., oxygen, O_2_) was included in this study. For this reason, we also introduced one more parameter (*K_o_*: half saturation constant for O_2_) to represent the limitation of O_2_ on the microbial processes sketched in Fig. 1. Similar to substrates, the saturation level of O_2_ is computed as *O*_2_/(*O*_2_+*K_o_*), where *O*_2_ denotes the concentration of oxygen. The simulated oxygen concentrations by Stolpovsky et al. [4] were used as an input to our model. We used the SCEUA algorithm to determine the six model parameters in addition to the initial value for active fraction (*r*_0_).

A summary of the seven parameters (one of them is *r*_0_) and their fitted values is presented in Table 2. The initial active fraction (*r*_0_) has a median of 0.925 with the 95% confidence interval (CI) of [0.628–1.000]. It means that a high *r*_0_ is required for this experiment, but not necessary to be 1.0 set by Stolpovsky et al. [4]. The model and data are not sensitive to *β* since its 95% CI covers a wide range from 0.001 to 1. The reason is that the experiment only lasts for a very short time (33 h) so the influence of low metabolic rate at dormant state is insignificant.

**Table 2:** MEND model parameter values used for simulation of the experiment described in Fig. 3 of Stolpovsky et al. (2011).

Parameter	Fitted Value*	Initial Range	Description
*m_R_*	0.032, [0.011–0.048]	0.001–0.1	Specific maintenance rate for active biomass (h^−1^)
*α*	0.099, [0.045–0.181]	0.001–0.50	*m_R_*/(*μ_G_*+*m_R_*), *μ_G_* is specific growth rate (h^−1^)
*K_s_*	3.110, [1.387–5.652]	0.1–9.0	Half-saturation constant for substrate (mg L^−1^)
*Y_G_*	0.573, [0.463–0.600]	0.2–0.6	Growth yield factor (–)
*K_o_*	0.0008, [0.0007–0.001]	0.005–0.1	Half-saturation constant for dissolved oxygen (mM)
*β*	0.351, [0.001–1.000]	0.001–1	Ratio of dormant maintenance rate to *m_R_*
*r*_0_	0.925, [0.628–1.000]	0–1	Initial fraction of active biomass (–)

*Medians and 95% confidence intervals of the fitted values from 100 optimization runs, i.e., 100 different random seeds are used for the stochastic optimization algorithm.

**Figure 4:**
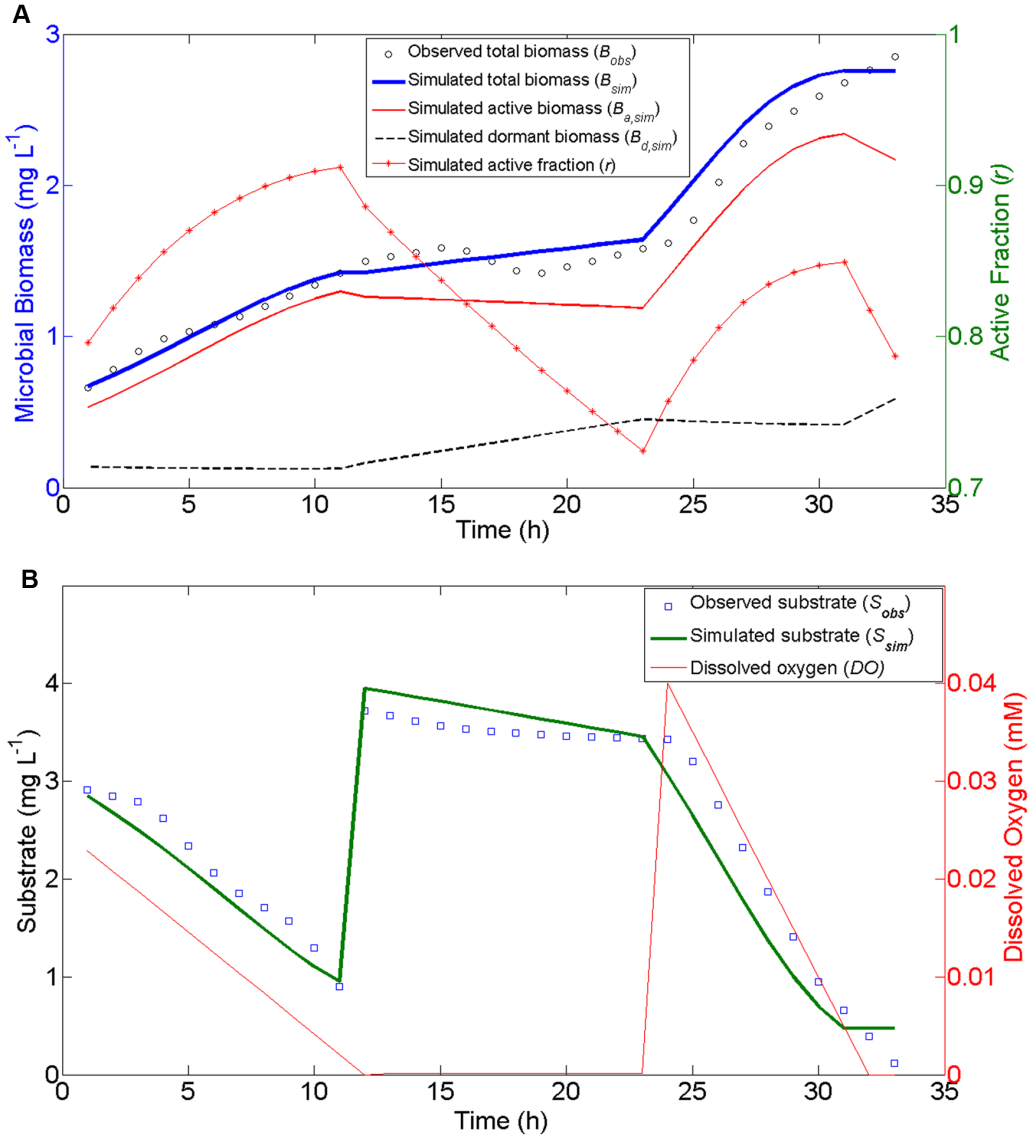
MEND model simulations against the experimental dataset used by Stolpovsky et al. (2011). (a) total live biomass, active and dormant biomass, and active fraction; (b) observed and simulated substrate concentration and prescribed O_2_ concentration. There are three manipulations on the substrate and oxygen: (1) at time 0, the substrate (3 mg/L) and O_2_ (0.025 mM) are added to the system; (2) after 12 h, the same amount of substrate is injected; (3) at 24 h, additional O_2_ (0.04 mM) is injected to the system. The observed concentrations of substrate and total biomass are hourly data interpolated from the original observations in Stolpovsky et al. (2011). We scaled the substrate concentrations (with units of mM in original data) to match the magnitude of biomass concentration in units of mg/L.

Fig. 4 shows that the simulated total biomass (*B*) and substrate (*S*) concentrations agree very well with the observations (the coefficients of determination are 0.98 and 0.78 for biomass in Fig. 4a and substrate in Fig. 4b, respectively). Our simulation results indicate that, under limited O_2_ between 12h and 24 h of the experiment, the active biomass decreases and the dormant biomass increases. As a result, the active fraction (*r*) declines from ca. 0.9 to 0.7 (Fig. 4a). For the same period Stolpovsky et al. [4] predicted a decrease of *r* from 1.0 to ca. 0, which means that all active biomass becomes dormant. Although there were not adequate measurements to confirm either prediction, our predicted changes in the active fraction (*r*) appear to be more reasonable during such a short experimental time period. This demonstration also shows that our model is capable of producing reasonable change in total, active, and dormant microbial biomass in response to substrate supply as well as an important forcing function (O_2_).

## Conclusions

We show that the physiological state index model (Equation 1) of Panikov [33] can be improved by eliminating the assumption that the steady state active fraction (*r^ss^*) approaches the substrate saturation level (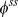). In particular, the model of Panikov [33] indicates that no active cells become dormant under insufficient substrate, which disregards the general nature of the strategy of dormancy in microorganisms when faced with unfavorable environmental conditions [5]. Our analysis also implies that the estimate of respiration rates under sufficient substrate by the physiological state index model is deficient. Pertaining to the switch function model, we argue that the switch function (*θ*) is also determined by the substrate (or combined with other impact factors) saturation level thus we would recommend using the switch function to modify the microbial uptake rate if the Gibbs energy change of the oxidation of substrate (Δ*G*) is tractable and the thermodynamic threshold (*G*_0_) and the steepness of the step function (*st*) are identifiable. Based on the generally accepted assumptions summarized from existing dormancy models, we postulate a synthetic microbial physiology component to account for dormancy. Both the steady state active fraction (*r^ss^*) and substrate saturation level (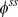) can be expressed as functions of two physiological indices: *α* and *β*. The index *α*  =  *m_R_*/(*μ_G_*+*m_R_*) is composed of *μ_G_* and *m_R_* denoting the maximum specific growth and maintenance rates, respectively, for active microbes. The index *β* represents the ratio of dormant to active maintenance rates. The value of *r^ss^* is no less than 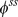, and is equal only under the condition of *β*→0, where they are both identical to *α*. The upper bound of *r^ss^* is ca. 0.8 at *α*→0.5 and *β*→1. The maximum *r^ss^* is ca. 0.5 if *β* (≤0.01) following the estimation of Anderson & Domsch [6]. It is evident that *r^ss^* could be attenuated further by other limiting factors. The application of the MEND microbial physiology model to an experimental dataset with intermittent substrate supply shows satisfactory model performance (the determination coefficients are 0.98 and 0.78 for microbial biomass and substrate, respectively). The case study on the SIGR dataset indicate that the exponentially-increasing respiration rates can only be used to determine *μ_G_* and *B_a0_* (initial active biomass), while the major parameters (*B*_0_, *r*_0_, *μ_G_*, *α*, *K_s_*) can be effectively determined using both exponentially-increasing and non-exponentially-increasing respiration rates.

In conclusion, the microbial physiology model presented here can be incorporated into existing ecosystem models to account for dormancy in microbially-mediated processes. We have illustrated the impacts of substrate and oxygen availabilities on the physiological states through this study. Other environmental factors, such as soil temperature and soil water potential, could also be introduced into this framework to affect the transformation processes between the two microbial compositions. The changes in the physiological states of microbes could further alter the microbially-driven carbon and nutrient dynamics in ecosystems. Traditional measures of microbial biomass include the entire microbial population, even though dormancy is an important evolutionary strategy for preservation of microbial genetics and function until conditions for growth and replication improve. Parameterizing microbial ecosystem models assuming the entire population is active could therefore lead to significant errors. The approach described here provides a tractable and testable method to include dormancy as a response to external forcing.

## Supporting Information

Appendix S1 **A summary of two-microbial-pool models.**
(DOCX)

Appendix S2 **Mathematical derivations.**
(DOCX)
