# Supplementary material for: Representation of Dormant and Active Microbial Dynamics for Ecosystem Modeling
Source: PLoS One. 2014 Feb 18;9(2):e89252. doi: 10.1371/journal.pone.0089252 (PMC3928434; doi:10.1371/journal.pone.0089252)
Supplement: Appendix S1 — A summary of two-microbial-pool models. (DOCX) [file pone.0089252.s001.docx]

**Representation of Dormant and Active Microbial Dynamics for Ecosystem Modeling**

Gangsheng Wang^1,2*^, Melanie A. Mayes^1,2^, Lianhong Gu^1,2^, [Christopher W. Schadt](mailto:schadtcw@ornl.gov)^1,3^

^1^Climate Change Science Institute, Oak Ridge National Laboratory, Oak Ridge, TN 37831-6301 USA

^2^Environmental Sciences Division, Oak Ridge National Laboratory, Oak Ridge, TN 37831-6301 USA

^3^Biosciences Division, Oak Ridge National Laboratory, Oak Ridge, TN 37831-6038 USA

**Appendix S1: A summary of two-microbial-pool models**

1. Transformation between active and dormant states

| Reference | Model description | Equation # |
| --- | --- | --- |
| [[1](#_ENREF_1)] | $B_{a\to d}^{N}=\left\{\begin{matrix} -\gamma_{d\to a}\cdot B_{d}, & \text{if }m\cdot B_{a}-S^{o}<0 \\ 0 & \text{if 0}\leq m\cdot B_{a}-S^{o}\leq\rho\cdot B_{a} \\ B_{a}-S^{o}/(\rho+m), & \text{if }m\cdot B_{a}-S^{o}>\rho\cdot B_{a} \end{matrix}\right.$ | (S1-1) |
| [[2](#_ENREF_2)] | $B_{a\to d}^{N}=\left\{\begin{matrix} (1-\delta)\cdot B_{a}-S^{o}/m & \text{, if }S^{\text{o}}<S^{R} \\ -(1-\delta)\cdot B_{d} & \text{, if }S^{\text{o}}\geq S^{R} \end{matrix}\right.$ $S^{R}=m(1-\delta)(B_{a}+B_{d})$   | (S1-2) |
| [[3](#_ENREF_3)] | $B_{a\to d}^{N}=\gamma_{a\to d}\cdot B_{a}-\gamma_{d\to a}\cdot B_{d}$ $\gamma_{a\to d}=\varepsilon_{a}/(S+K_{a})+v_{a}$ $\gamma_{d\to a}=\left\{\begin{matrix} v_{d}, & \text{if }S>K_{s} \\ 0, & \text{otherwise} \end{matrix}\right.$     | (S1-3) |
| [[4](#_ENREF_4)] | $B_{a\to d}^{N}=\left(1-\mu/\mu_{\max}\right)\cdot\gamma_{a\to d}\cdot B_{a}-\left(\mu/\mu_{\max}\right)\cdot\gamma_{d\to a}\cdot B_{d}$ | (S1-4) |
| [[5](#_ENREF_5)] | $B_{a\to d}^{N}=\left(1-R\right)\cdot B_{a}-R\cdot B_{d}$ $R=R_{\max}\cdot\exp\left\{-(1-W_{f})\cdot[(E_{L}^{*}-E_{L})/tol]^{2}-W_{f}\cdot[(E_{R}^{*}-E_{R})]^{2}\right\}$   | (S1-5) |
| [[6](#_ENREF_6)] | $B_{a\to d}^{N}=v\cdot\left\{\left[1-J\left(W-W_{ad}\right)\right]\cdot B_{a}-J\left(W-W_{ad}\right)\cdot B_{d}\right\}$ $J\left(W-W_{ad}\right)=0.5\cdot\tanh\left[(W-W_{ad})/s\right]$   | (S1-6) |
| [[7](#_ENREF_7)] | $B_{a\to d}^{N}=\gamma_{a\to d}\cdot B_{a}-\left(1-p\right)\cdot\left(B_{d}/B\right)\cdot B_{d}$ | (S1-7) |

**Variables and Parameters**:

*B_a_* : active biomass (mg C, mg C cm^−3^, or mg C g^−1^ soil, hereinafter referred to as mg C g^−1^);

*B_d_* : dormant biomass (mg C g^−1^);

$B_{a\to d}^{N}$: net transformation of *B_a_* to *B_d_* (mg C h^−1^, mg C cm^−3^ h^−1^, or mg C g^−1^ h^−1^, hereinafter referred to as mg C g^−1^ h^−1^);

*E_L_* and *E_L_*^*^: local environmental cue and its optimum;

*E_R_* and *E_R_*^*^: regional environmental cue and its optimum;

*J*(*W* − *W_ad_*): probability for dormant bacteria to become active;

*K_a_* (<*K_s_*): half-saturation constant for the conversion of active to dormant state (mg C g^−1^);

*K_s_*: half-saturation constant (mg C g^−1^);

*m*: maintenance respiration rate (d^−1^ or h^−1^, hereinafter referred to as h^−1^);

*p*: (1 – *p*) represents the probability of reactivation of dormant microbes;

*R* and *R*_max_: resuscitation rate and maximum resuscitation rate (h^−1^);

*s*: denote the steepness of the transformation function *J*(*W* − *W_ad_*) in Bär *et al*. [[6](#_ENREF_6)];

*S*: substrate concentration (mg C g^−1^);

*S^o^*: potential offer of microbial substrate (mg C g^−1^ h^−1^);

*S^R^*: maintenance respiration requirement (mg C g^−1^ h^−1^);

*tol*: environmental tolerance;

*v*: transformation rate between active and dormant states (h^−1^);

*v_a_* and *v_d_*: rate constants (h^−1^);

*W*: a stress field, e.g., soil humidity close to the surface in Bär *et al*. [[6](#_ENREF_6)];

*W_ad_*: a critical value of the stress field, below which active bacteria incline to become dormant and vice versa;

*W_f_*: weighting of local vs. regional environmental cues;

*ε_a_*: rate constant (mg C h^−1^);

*γ_a_*_→_*_d_* and *γ_d_*_→_*_a_*: transformation rates of active to dormant state and dormant to active state (h^−1^);

*μ* and *μ*_max_: specific growth rate and maximum specific growth rate (h^−1^);

*ρ*: threshold rate that is set to 0.01 d^−1^.

*δ*: microbial mortality rate (h^−1^);

1. Switch function model

The switch function [[8](#_ENREF_8)], i.e., determining the fraction (*θ*) of active biomass that uptakes substrate (e.g., DOC), follows a smoothed step function adapted from Fermi-Dirac statistics:

$dB_{a}/dt=Y_{eff}\cdot\theta\cdot\mu(S,TEA)\cdot B_{a}-m_{a}\cdot B_{a}-(1-\theta)\cdot v_{deact}\cdot B_{a}+Y_{react}\cdot I_{d}\cdot\theta\cdot v_{react}\cdot B_{d}$ (S1-8a)

$dB_{d}/dt=-m_{d}\cdot B_{d}+(1-\theta)\cdot v_{deact}\cdot B_{a}-I_{d}\cdot\theta\cdot v_{react}\cdot B_{d}$ (S1-8b)

$dI_{d}/dt=I_{d}\cdot\left[k_{incr}\cdot\theta\cdot(1-\theta_{I})-k_{decr}\cdot(1-\theta)\right]$ (S1-8c)

$\theta=\frac{1}{\exp\left(\frac{-G+G_{0}}{st\cdot G_{0}}\right)+1}$ (S1-8d)

$G=\Delta G\cdot\frac{1}{B_{a}}\cdot\left.\frac{dS}{dt}\right|_{r=1,Y_{G}=0}=-\Delta G\cdot\mu(S,TEA)$ (S1-8e)

where *B_a_* and *B_d_* are the active and dormant biomass, respectively; *v_react_* and *v_deact_* are the specific rates for reactivation and deactivation, respectively; *Y_react_* (0.1–1) denotes the reactivation yield; *Y_eff_* is the effective growth yield; *m_a_* and *m_d_* represent the maintenance coefficient for *B_a_* and *B_d_*, respectively; *I_d_* (0–1) represents the ‘depth’ of dormancy; *k_incr_* and *k_decr_* (0.05–0.5 h^−1^) are the first-order rate constants describing the increase and decrease of *I_d_* under favorable (*θ*→1) or unfavorable (*θ*→0) conditions, respectively; *θ_I_* is selected to ensure *I_d_* ≤1; *G* is the maximum rate of Gibbs energy release per unit biomass; and *G*_0_ (0.1–25 kJ mol^−1^ biomass h^−1^) represents a corresponding minimum threshold value; ∆*G* is the Gibbs energy change of the oxidation of substrate (e.g., oxidation of DOC into CO_2_); *st* is dimensionless and denotes the steepness of the step function; *μ*(*S*, *TEA*) is the growth rate as a function of concentrations of substrate (*S*) and terminal electron acceptor (*TEA*). Stolpovsky *et al*. [[8](#_ENREF_8)] arbitrarily assigned a value of 0.1 to *st* that leads to a narrow but finite “switching zone”. The value of ∆*G* is controlled by the concentration of substrate and products as well as the terminal electron acceptor (TEA).

**References:**

1. Hunt HW (1977) A simulation model for decomposition in grasslands. Ecology 58: 469-484.

2. Gignoux J, House J, Hall D, Masse D, Nacro HB, et al. (2001) Design and test of a generic cohort model of soil organic matter decomposition: the SOMKO model. Global Ecology and Biogeography 10: 639-660.

3. Ayati BP (2012) Microbial dormancy in batch cultures as a function of substrate-dependent mortality. Journal of Theoretical Biology 293: 34-40.

4. Konopka A (1999) Theoretical analysis of the starvation response under substrate pulses. Microbial Ecology 38: 321-329.

5. Jones SE, Lennon JT (2010) Dormancy contributes to the maintenance of microbial diversity. Proceedings of the National Academy of Sciences 107: 5881-5886.

6. Bär M, Hardenberg J, Meron E, Provenzale A (2002) Modelling the survival of bacteria in drylands: the advantage of being dormant. Proceedings of the Royal Society of London Series B: Biological Sciences 269: 937-942.

7. Locey KJ (2010) Synthesizing traditional biogeography with microbial ecology: the importance of dormancy. Journal of Biogeography 37: 1835-1841.

8. Stolpovsky K, Martinez-Lavanchy P, Heipieper HJ, Van Cappellen P, Thullner M (2011) Incorporating dormancy in dynamic microbial community models. Ecological Modelling 222: 3092-3102.
