# Supplementary material for: Representation of Dormant and Active Microbial Dynamics for Ecosystem Modeling
Source: PLoS One. 2014 Feb 18;9(2):e89252. doi: 10.1371/journal.pone.0089252 (PMC3928434; doi:10.1371/journal.pone.0089252)
Supplement: Appendix S2 — Mathematical derivations. (DOCX) [file pone.0089252.s002.docx]

**Representation of Dormant and Active Microbial Dynamics for Ecosystem Modeling**

Gangsheng Wang^1,2^, Melanie A. Mayes^1,2^, Lianhong Gu^1,2^, [Christopher W. Schadt](mailto:schadtcw@ornl.gov)^1,3^

^1^Climate Change Science Institute, Oak Ridge National Laboratory, Oak Ridge, TN 37831-6301 USA

^2^Environmental Sciences Division, Oak Ridge National Laboratory, Oak Ridge, TN 37831-6301 USA

^3^Biosciences Division, Oak Ridge National Laboratory, Oak Ridge, TN 37831-6038 USA

# Appendix S2: Mathematical derivations

1. Derivation of Equation 4 based on Panikov [[1](#_ENREF_1),[2](#_ENREF_2)]:

From Equation 1, it follows that

$\frac{dB_{a}}{dt}=\frac{d(rB)}{dt}=r\frac{dB}{dt}+B\frac{dr}{dt}=r\frac{dB}{dt}+B\cdot\frac{1}{B}\frac{dB}{dt}(\varphi-r)=\varphi\frac{dB}{dt}$ (S2-1)

1. Derivation of Equation 12e: change of rate of active fraction (*r*)

$\begin{matrix} \frac{dr}{dt}=\frac{1}{B}\left(\frac{dB_{a}}{dt}-r\frac{dB}{dt}\right) \\ =\frac{1}{B}\left\{(\varphi/\alpha-1)\cdot m_{R}\cdot B_{a}-(1-\varphi)\cdot m_{R}\cdot B_{a}+\varphi\cdot m_{R}\cdot B_{d}-r\left[(\varphi/\alpha-1)\cdot m_{R}\cdot B_{a}-\left(\beta\cdot m_{R}\right)\cdot B_{d}\right]\right\} \\ =m_{R}\left\{(\varphi/\alpha-1)\cdot r-(1-\varphi)\cdot r+\varphi\cdot(1-r)-r\left[(\varphi/\alpha-1)\cdot r-\beta\cdot(1-r)\right]\right\} \\ =m_{R}\cdot\left[(\varphi-r)+(\varphi/\alpha+\beta-1)\cdot r\cdot(1-r)\right] \end{matrix}$ (S2-2)

1. Steady state solution to the synthetic microbial physiology model (Equation 12)
2. *β* > 0

$S^{ss}=K_{s}\frac{\alpha-\beta+3\alpha\beta+\sqrt{C^{2}+8\alpha\beta}}{2(1-\alpha)(1+\beta)}$ (S2-3a)

$B^{ss}=\frac{Y_{G}I_{S}}{m_{R}}\frac{\alpha\left(1-\beta\right)^{2}+3\beta+\beta^{2}-(1-\beta)\sqrt{C^{2}+8\alpha\beta}}{4\beta^{2}}$ (S2-3b)

$B_{a}^{ss}=\frac{Y_{G}I_{S}}{m_{R}}\frac{C+\sqrt{C^{2}+8\alpha\beta}}{4\beta}$ (S2-3c)

where $C=\alpha(\beta-1)+\beta$

$r^{ss}=B_{a}^{ss}/B^{ss}$ (S2-3d)

$\varphi^{ss}=S^{ss}/\left(S^{ss}+K_{s}\right)$ (S2-3e)

1. *β* = 0

$S^{ss}=K_{s}\cdot\alpha/(1-\alpha)$ (S2-4a)

$B^{ss}=I_{s}\cdot Y_{G}/(\alpha\cdot m_{R})$ (S2-4b)

$B_{a}^{ss}=I_{s}\cdot Y_{G}/m_{R}$ (S2-4c)

$r^{ss}=\varphi^{ss}=\alpha$ (S2-4d)

1. *β* = 1

$S^{ss}=K_{s}\frac{4\alpha-1+\sqrt{1+8\alpha}}{4(1-\alpha)}$ (S2-5a)

$B^{ss}=I_{s}\cdot Y_{G}/m_{R}$ (S2-5b)

$B_{a}^{ss}=\frac{I_{s}\cdot Y_{G}}{m_{R}}\cdot\frac{1+\sqrt{1+8\alpha}}{4}$ (S2-5c)

$r^{ss}=\frac{1+\sqrt{1+8\alpha}}{4}$ (S2-5d)

$\varphi^{ss}=\frac{4\alpha-1+\sqrt{1+8\alpha}}{3+\sqrt{1+8\alpha}}$ (S2-5e)

1. Derivation of Equation 14.

Simplification of Equations 12(a–e) under conditions of (i) excess of substrate (*ϕ*→1), (ii) $I_{s}$= 0, and (iii) the maintenance respiration of dormant microbes [$\left(\beta\cdot m_{R}\right)\cdot B_{d}$] may be negligible compared to the growth and maintenance respiration of active microbes.

$dS/dt=-\frac{1}{Y_{G}}\cdot(\mu_{G}+m_{R})\cdot(r\cdot B)$ (S2-6a)

$dB/dt=\mu_{G}\cdot(r\cdot B)$ (S2-6b)

$dr/dt=\mu_{G}\cdot r\cdot(1-r)+m_{R}\cdot(1-r)$ (S2-6c)

Equations S2-6(a–c) are integrated for initial conditions, i.e., *S* = *S*_0_, *B* = *B*_0_ and *r*=*r*_0_ at *t* = 0:

$S(t)=S_{0}-\frac{B(t)-B_{0}}{Y_{G}(1-\alpha)}$ (S2-7a)

$B(t)=B_{0}r_{0}\cdot e^{\left(1/\alpha-1\right)m_{R}t}+B_{0}(1-r_{0})\cdot[\alpha\cdot e^{\left(1/\alpha-1\right)m_{R}t}+(1-\alpha)\cdot e^{-m_{R}t}]$ (S2-7b)

$r(t)=\frac{[r_{0}+\alpha(1-r_{0})]\cdot e^{\left(m_{R}/\alpha\right)\cdot t}-\alpha(1-r_{0})}{[r_{0}+\alpha(1-r_{0})]\cdot e^{\left(m_{R}/\alpha\right)\cdot t}+(1-\alpha)(1-r_{0})}$ (S2-7c)

The CO_2_ production rate during the exponential growth stage, *v*(*t*), is derived as an explicit function of *t*:

$\begin{matrix} v(t)=\frac{dCO_{2}}{dt}=\frac{1-Y_{G}}{Y_{G}}(m_{R}/\alpha)\cdot(r\cdot B) \\ =\frac{B_{0}(1-Y_{G})}{Y_{G}}\left\{\left[(m_{R}/\alpha)\cdot r_{0}+m_{R}\cdot(1-r_{0})\right]\cdot e^{\left(1/\alpha-1\right)m_{R}t}-\left[m_{R}\cdot(1-r_{0})\right]\cdot e^{-m_{R}t}\right\} \end{matrix}$ (S2-7d)

If *m_R_* << *μ_G_* (i.e., $\alpha$→0), Equations S2-7(a–d) are further simplified to

$S(t)=S_{0}-\frac{B(t)-B_{0}}{Y_{G}}$ (S2-8a)

$B(t)=B_{0}r_{0}\cdot e^{\mu_{G}t}+B_{0}(1-r_{0})$ (S2-8b), similar to Equation 11 in [[3](#_ENREF_3)]

$r(t)=\frac{r_{0}}{r_{0}+(1-r_{0})\cdot e^{-\mu_{G}t}}$ (S2-8c), similar to Equation 10 in [[3](#_ENREF_3)]

$v(t)=\frac{B_{0}r_{0}(1-Y_{G})}{Y_{G}}\mu_{G}\cdot e^{\mu_{G}t}$ (S2-8d), different from Equation 13 in [[3](#_ENREF_3)] but identical to Equation 7 in [[4](#_ENREF_4)]

**References:**

1. Panikov NS (1996) Mechanistic mathematical models of microbial growth in bioreactors and in natural soils: explanation of complex phenomena. Mathematics and Computers in Simulation 42: 179-186.

2. Panikov NS (1995) Microbial growth kinetics. London, UK: Chapman & Hall. 378 p.

3. Panikov NS, Sizova MV (1996) A kinetic method for estimating the biomass of microbial functional groups in soil. Journal of Microbiological Methods 24: 219-230.

4. Colores GM, Schmidt SK, Fisk MC (1996) Estimating the biomass of microbial functional groups using rates of growth-related soil respiration. Soil Biology and Biochemistry 28: 1569-1577.
